# Towards Drug Delivery Control Using Iron Oxide Nanoparticles in Three-Dimensional Magnetic Resonance Imaging

**DOI:** 10.3390/nano11081876

**Published:** 2021-07-22

**Authors:** Mohammed Almijalli, Ali Saad, Khalid Alhussaini, Adham Aleid, Abdullatif Alwasel

**Affiliations:** Department of Biomedical Technology, College of Applied Medical Sciences, King Saud University, P.O. Box 10219, Riyadh 11433, Saudi Arabia; malmijalli@ksu.edu.sa (M.A.); kalhussaini@ksu.edu.sa (K.A.); adaleid@ksu.edu.sa (A.A.); alwasel@ksu.edu.sa (A.A.)

**Keywords:** drug delivery, MRI, FCM algorithm, nanoparticle mining, nanomedicine

## Abstract

The purpose of this paper was to detect and separate the cluster intensity provided by Iron oxide nanoparticles (IO-NPs), in the MRI images, to investigate the drug delivery effectiveness. IO-NPs were attached to the macrophages and inserted into the eye of the inflamed mouse’s calf. The low resolution of MRI and the tiny dimension of the IO-NPs made the situation challenging. IO-NPs serve as a marker, due to their strong intensity in the MRI, enabling us to follow the track of the macrophages. An image processing procedure was developed to estimate the position and the amount of IO-NPs spreading inside the inflamed mouse leg. A fuzzy Clustering algorithm was adopted to select the region of interest (ROI). A 3D model of the femoral region was used for the detection and then the extraction IO-NPs in the MRI images. The results achieved prove the effectiveness of the proposed method to improve the control process of targeted drug delivered. It helps in optimizing the treatment and opens a promising novel research axis for nanomedicine applications.

## 1. Introduction

Significant limitations in drug administration and delivery procedures is the inability to target drug amounts and spread in the ROI [1]. Magnetic nanoparticles (MNPs) retain exclusive magnetic properties and the capability of operating at the cell and the molecule level of biological interactions. Recent developments in nanotechnology involved the appearance of MNPs as an operational method of drug delivery schemes. They can be engaged via outwardly applied magnetic fields and their functional properties can be explicitly modified to the purpose [2]. This makes MNPs a smart portal to study drug delivery carriers for MRI in [3]. Amongst diverse cell types, macrophages are capable of moving to sites of inflammation, infection, and tissue degeneration; this makes them appealing vehicles for delivering diagnostic contrast agents or therapeutic medicines [4]. Diverse environmental variables inside the body will cause macrophages to take different actions. This chemokine-driven process, which can occur in vivo and can also be replicated in vitro, is known as macrophage polarization. At least two opposing polarization states with separate roles exist in macrophages [5]. Traditional activated macrophages, also known as the M1 subpopulation, have a pro-inflammatory function and can be activated by IFN-alone or in combination with microbial stimuli (i.e., bacterial lipopolysaccharides, LPS). Otherwise M2 macrophages, which are induced by IL-4 and IL-13, immune complexes, IL-10, and glucocorticoids, have immune-modulating properties and endorse wound healing and angiogenesis. Nevertheless, available data do not completely clarify in what way infection induces modulation of M1 and M2 phenotypes, and whether distinct macrophage subsets predominate after an inflammation process. While it is widely established that macrophages migrate along a chemokine gradient to the site of injury [6], the dynamics driving the migration of M1 or M2 subsets are yet to be understood [7]. In [8], the authors use high-resolution MRI cell tracking to trace the trafficking of variably polarized macrophages in a murine model of local inflammation. Ex-vivo polarization of bone marrow-derived macrophages was performed, and various subsets of macrophages were tagged with AMNPs. The authors measured AMNP uptake in various macrophage subsets and assessed the impact of magnetic labeling on their phenotypic expression. Inflammation-bearing animals were given magnetically-tagged macrophage subpopulations, intravenously, and their infiltration to the site of inflammation was monitored in real-time using a commercially available high-resolution cryogenic probe that permitted single cell detection [8].

Alternatively, high-resolution MRI is, primarily, very appropriate for image-guided drug delivery because of its high spatial resolution and ability to acquire quantitative measurements during therapy. MRI-guided drug delivery may facilitate minimally invasive image-guided diagnosis and therapies [1]. 

Image partition is the main research theme as the gateway in pattern or object recognition and scene analysis. Segmentation splits an image into its distinct constituents. Current segmentation eases the analysis of images. We briefly describe some of the recent work conducted by researchers on clustering techniques via fuzzy clustering algorithms. Automatic segmentation is a procedure that provides exclusive information on the intensity disparity of neighbor pixels in medical images. It is useful to identify an abnormal tumor or suspect object in images [9]. Segmentation is generally practiced in radiotherapy, surgery, and neurosurgery, in addition to several other medical specialties [10,11]. However, this recognition technique lacks precise localization and findings of nanoparticle clusters and shapes.

The eight types of medical image segmentation methods available in the literature are threshold approaches, clustering approaches, classifiers, area increasing approaches, Artificial Neural Networks (ANNs), Markov Random Field (MRF), and deformable models [12]. Among these techniques, cluster-based approaches have received a lot of attention in the medical imaging research community. The crisp clustering scheme and the fuzzy clustering scheme were among the clustering methods used by researchers. Each has its own distinct characteristics [13].

Restricted spatial resolution, low contrast, overlapping intensities, noise, and intensity variations inhomogeneity make crisp clustering a challenging task for images in many real-world scenarios. Fuzzy clustering system, on the other hand, has been extensively studied as a soft segmentation tool and has been effectively realistic in many medical image segmentation methods [14].

In [11], a report on segmenting 3D-MRI brain images into various tissue types is described. The k-means clustering algorithm was used to classify gray matter, white matter, CSF (cerebrospinal fluid), and other pathological tissues in the brain. The work in [15] demonstrates how to identify brain tumors using MRI and segment them using the k-means and FCM algorithms to determine their exact position and scale. The clustering algorithms demonstrate the simple brain structure and distinguish between benign and malignant tumors in the brain. In [15,16], a method for detecting the progression of brain tumors using slice MRI images was suggested.

The staging of a brain tumor involves the classification of irregular tissue masses. Pathological brain tissue comes in several shapes and sizes, making diagnosis and assessment difficult. The results in [15] compare the output of the k-means algorithm to the fuzzy c-means algorithm used for segmenting MRI brain images [16,17].

In [17], for the segmentation of medical images, a robust segmentation technique based on the FCM algorithm is developed and exploits histogram. The algorithm starts by eliminating noise from the images before moving on to segmentation. Denoising is achieved using sparse 3D transform–domain collaborative filtering. The histogram is used to initialize the parameters of the FCM to avoid convergence in local minima. The objective function integrates spatial likelihood to increase the algorithm’s noise tolerance. Two forms of spatial information are included in the proposed segmentation technique. A priori likelihood is the first, and fuzzy spatial information is the second. To allocate a noisy pixel to a cluster that includes a large number of noisy pixels in its neighborhood, the membership function integrates a priori probability. Fuzzy spatial probability is used in the membership function, and a pixel has a higher membership value to a cluster if its neighboring pixels have a high membership value to that cluster [18]. This method converges faster than traditional FCM and achieves accurate segmentation regardless of noise levels. To allocate a noisy pixel to a cluster that includes a large number of noisy pixels in its neighborhood, the membership function integrates a priori probability. This approach achieves correct segmentation accuracy, regardless of noise levels, and converges faster than conventional FCM.

The FCM algorithm can help distinguish abnormalities in MRI brain images, such as benign and malignant brain tumors and other diseases, as well as recognize any abnormal particles in the picture [17]. The FCM has also been used to determine where the affected brain area is located [18]. In [19], they studied anticancer drug sensitivity in human lung cancer using the FCM strategy.

In [20], a k-means algorithm was used for extraction of NPs from HR-MRI images. As FCM has proven to be the more realistic segmentation method [21], this paper proposes an FCM based method for segmentation of 2D slices. A novel method of extracting IO-NPs pixels from ROI by using morphological operation in MATLAB is proposed for drug delivery monitoring in lesions or tumors for therapeutic purposes. The IO-NPs were collected in stages from the HR-MRI images of the mouse calf. The FCM algorithm was used to segment the images into seven groups after applying a median filter to the entire stack of 50 images for background correction and noise filtering. The NPs were then isolated from the calf tissue using Otsu’s binarization method [22,23]. Finally, image processing techniques were proposed to extract IO-NPs from ROI. A 3D processing was also proposed to extract nanoparticles from a 3D map that displayed the distribution of NPs across the entire calf. The extraction of NPs from the dissemination of 2D images and their spread in 3D images demonstrated great potential in the current analysis.

## 2. Materials and Methods

### 2.1. Materials

In this paper, we use the same data as the one used in [8]. A description of nanoparticle synthesis and characterization, as well as labeling of macrophages and statistics, are presented in [8]; IO-NPs labeled macrophages are inoculated in the eye of the mouse. Inflammation was made in the right calf muscle by intramuscular injection of 100 mL PBS suspension comprising 50 mg *E. coli*-derived LPS (Sigma Aldrich, Lyon, France) under 1.5% isoflurane anesthesia. Bacterial LPS injection was directed 4 h prior to saline, Io-NPs or macrophage transfusion [8]. For clinical trials, MR imaging study, a 40-cm bore scale, actively-shielded, 4.7 Tesla Bruker magnets interfaced with ParaVision software (Bruker Biospin GmbH, Rheinstetten, Germany) was used.

The cryogenic surface probe was used to reach high-resolution MRI (HR-MRI) (CryoProbe^TM^, Bruker).

Three-dimensional (3D) liability biased echo sequence (TR/TE = 20/5 ms, flip angle = 25°), 2 averages, and a pixel resolution of 50 × 50 × 50 μm^3^ for a total acquisition time of 10 min was applied to an identical area of the muscle for all of the mice.

**Figure 1:**
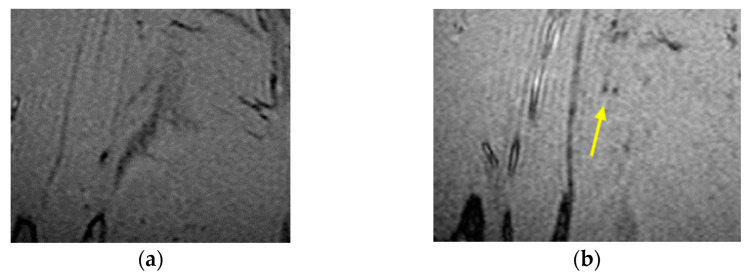
MR images of the mouse calf region (**a**) lacking IO-NPs and (**b**) with IO-NPs, as black dots in the image pointed by yellow arrow.

The technique was tested on a series of MRI images of the leg comprising nanoparticles using the MATLAB and ImageJ program and applied to grayscale images of size 300 × 300 pixels. The format used was (.tiff). The first set of data contained a stack of 50 images of a mouse calf with IO-NPs and a second set of 50 images of identical sizes, but no IO-NPs. The aim was to calibrate and validate the consistency of the IO-NP identification method. Figure 1 shows the two slices with and without IO-NPs.

### 2.2. Image Pre-Processing 

#### 2.2.1. Noise Filtering

Image processing techniques may assist in improving segmentation accuracy and separating nanoparticles from other tissues, resulting in a precise and detailed nanoparticle extract. The accuracy of the images and data associated with the results in MRI are compromised by noise background and inconsistency. Raw data are pre-processed to eliminate noise in order to increase efficiency and the segmentation scheme [24]. The first step in medical image segmentation is pre-processing, which involves enhancing the interpretability or interpretation of information in images. Furthermore, the medical MR images are enhanced with finer details, and noise is minimized [10]. A local median filter is used to eliminate noise from images; the clue behind the median filter is to run through the image pixel by pixel, replacing each pixel with the median of neighboring (5 × 5) pixels, which will make the pixel more harmonious with its neighbors. Median filtering is a nonlinear process that decreases image noise while retaining image edges; it is more efficient than averaging linear filter [24].

#### 2.2.2. Background Subtraction

Background subtraction was achieved on a stack of 50 images. The ‘‘Rolling Ball’’ (Sternberg’s 1983) algorithm was used since it eliminated flat continuous backgrounds from images, which is most appropriate for MRI images. A local background value was determined for every pixel by averaging over a very large ball around the pixel. This value was hereafter subtracted from the original image, removing large spatial variations of the background intensities. Classic values of the radius is normally between 0.2 and 5 for 16-bit images. 

### 2.3. Image Segmentation

The main aim of image segmentation is to remove different features from an image that can be combined or separated, in order to locate the object of interest [24]. Image segmentation is useful for locating and exploring any object in a picture, as well as selecting borders (lines, curves, etc.). The segmentation is based on the image’s basic properties, interruptions, and similarities. Splitting and subdivision are both based on sudden shifts in the image’s intensity or gray levels [24]. The effect of image segmentation is a collection of segments that comprise the entire image collectively. In the k-means method, clusters are well predefined, and highly dependent on the initial identification of elements representing the clusters. FCM is a generalization of the k-means that overcomes this inconvenience of the dependency of the initial choice of cluster centers. 

#### 2.3.1. Fuzzy Clustering Segmentation Method 

Fuzzy clustering is an automated segmentation method for image analysis in which pixels in one cluster are more identical to those in other clusters [25,26,27,28]. Since it considers the data distribution in a class to be spherical, it is a generalization of the *k*-means algorithm [26,29]. This algorithm is characterized by a positive integer with a constant value for “*k*” clusters.

Meanwhile, the FCM considers a class’s data distribution to be elliptical, which is more practical, and confirms its reputability and heftiness.

Equation (1) represents the function to be minimized in order to obtain optimal segmentation

(1) $$J_{m}\left(U,V\right)=\sum_{j=1}^{N}\sum_{i=1}^{C}u_{ij}^{m}d^{2}\left(x_{j},v_{i}\right)$$

where X = {*x*_1_, *x*_2_, …, *x*_j_, …, *x_N_*} is a p × N data matrix, where, p signifies the dimension of each *x_j_* ‘‘feature’’ vector, and N denotes the number of feature vectors (pixel numbers in the image). ‘C’ is the number of clusters or classes. *U_ij_* included in U (p, N, C) is the membership function of vector *x_j_* to the *j^th^* cluster, which satisfies *u_ij_* is between (0, 1).

uij=1∑k=1C(d(xj,vi)d(xj,vi))2m−1

The cluster feature center is denoted by a matrix *V* = {*v*_1_, *v*_2_, *v_i_*, …, *v*_C_} of dimension C.

(2) $$v_{i}=\frac{\sum_{j=1}^{N}{\left(u_{ij}\right)}^{m}x_{j}}{\sum_{j=1}^{N}{\left(u_{ij}\right)}^{m}}\;\;\;i=1,\;2,\;\ldots ,\;C$$

*m* is a weighting exponent on each fuzzy membership, which controls the degree of fuzziness. *d(x_j_, v_i_)* is a measurement of similarity between *x_j_* and *v_i_*.

$$d^{2}\left(x_{j},v_{i}\right)=\|x_{j}-v_{i}\|$$

||.|| is a Euclidian distance in this study.

#### 2.3.2. Implementation of Fuzzy Clustering Algorithm

The fuzziness level was set to two. Variations in the degree of fuzziness were found to have no major impact on segmentation performance. The pixel intensities are represented by the feature vector ‘X’ in the MR image, so *p* = 1. The mean intensity of classes, derived from the histogram-driven initialization, is represented by cluster centers in V, and the number of classes in the pre-processed MR image is represented by C. *J_m_* (U,V) was improved iteratively by updating U and V until the algorithm converged to a very small number ε, where ε was chosen to be 0.0001.

The FCM algorithm was implemented using MATLAB software in this experimental analysis. We applied the algorithm steps to the dataset as follows:

- Chose the number of clusters: un this analysis, the number of clusters was set to seven (*k* = 7). To make the iteration process go faster, the initial centers (*c_j_*) were chosen to be equidistant from one another.
- Computed the distance between *x_i_* and each cluster center *c_j_*, then assigned each pixel to the cluster with the shortest distance between them using Equation (1) as the function to be minimized. The distance between data ‘*i*’ and cluster ‘*j*’ is D(*x_i_*, *c_j_*).
- For each of the seven clusters, we calculated the new cluster centroid (*c_j_*). The new cluster centroid was determined using Equation (2): where, *N* and $u_{ij}$ are the number and value of member objects within each cluster, respectively.
- Steps 2 and 3 should be repeated until the mean value convergence is achieved and the center does not change.

#### 2.3.3. Post-Processing to Extract IO-NPs from 2D Images

After segmentation, we selected the class where the inflamed area was in to explore the nanoparticles. The MATLAB function bwareafilt was used for this purpose [30]. Next, the ROI was transformed into a binary image using Otsu’s method in ImageJ software [22], the threshold was chosen according to study from the original set of data without IO-NPs to eliminate noise, and any artifacts due to hemoglobin and/or particle motion in MRI images. Then, the identification of the NPs in this region was obtained using morphological operations to extract the NPs from the mouse calf residual structure. Connected components and size analysis were also used to extract the IO-NPs from the 2D slice.

### 2.4. Nanoparticles Detection

A 3D reconstruction and visualization was established using 3D plugins in ImageJ software [23]. In this step, we benefited from the methods applied to the tumor detection and area calculation in MRI image of the human brain using image processing techniques [31,32,33,34,35,36].

Accurate nanoparticle extraction is a critical task due to its complex concentration in the inflamed region of the leg. After the segmentation of the leg region using the FCM algorithm, the nanoparticles were extracted as follows. First, the images were converted to binary black and white pixels as described in 2.3.3 [22]. The binary picture, with only two levels, black background, and white objects, included the targeted IO-NPs and mouse calf leg structures. The mouse leg structures, such as bone and muscles, should be removed to reveal only the NPs in the resulting view. First, we used morphological operations on the binary image to create a mask containing only mouse calf structure and cleared all outlying pixels that were considered noise. In order to create a mask containing only the calf structure at the same dimension as the original image, an erosion consisting of a two-pixel radius called “opening” was applied, followed by dilation of the same radius called “closing”. The ‘‘analyze particles” method in ImageJ was used for this purpose, with parameters set to provide images with only the linear structure (no NPs). This was based on the assumption that IO-NPs, or aggregation of it, did not form linear shapes. On the other hand, IO-NP clusters may take on spherical or elliptical shapes. To set up the parameters of the particle analysis tool, we used, as a reference, a control stack of MRI images of the mouse calf that did not include IO-NPs. The IO-NPs distribution was then obtained by subtracting the input image from the resulting image of the particle analysis.

## 3. Results

### 3.1. Results IO-NPs Extraction from 2D Images 

**Figure 2:**
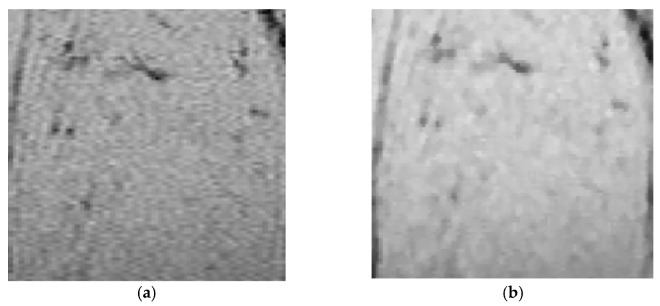
Pre-processing: (**a**) original image, (**b**) pre-processing image.

**Figure 3:**
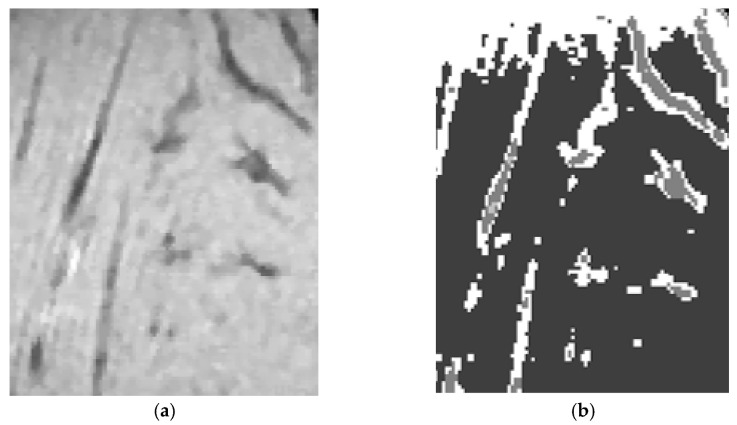
(**a**) Input 2D image, (**b**) image segmented by fuzzy clustering.

The output of the pre-processing (filtering) step is shown in Figure 2b. The filtered images were taken as the input of the segmentation by the FCM algorithm and applied to find the image classes. Figure 3b shows the results of the segmented image by the FCM algorithm. The classes are presented using gray levels where the central part of the segmented image represents the ROI.

### 3.2. Results of IO-NPs Extraction from Slices of 3D Model 

**Figure 4:**
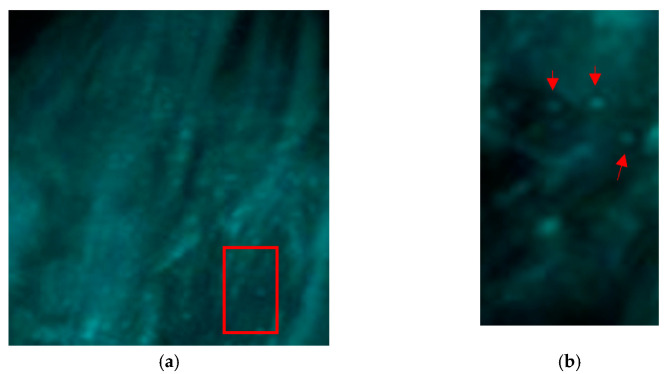
Shows a 3D view of the mouse calf, in (**a**), 3D visualization of the mouse calf, the rectangular shape is zoomed and presented in (**b**). Red arrows pointing to the blue ball-shaped particles representing clusters of IO-NPs.

Figure 4 shows the 3D view of the mouse calf. Figure 4a shows the 3D view of the calf after background subtraction. A red rectangle from the ROI is zoomed-in (in Figure 4b). The red arrows point to the blue balls representing intensities of clusters of IO-NPs. By seeing the distribution of the blue balls in the ROI, it provides us with a qualitative visual estimation of the existence and distribution of the IO-NPs in the inflamed calf region.

**Figure 5:**
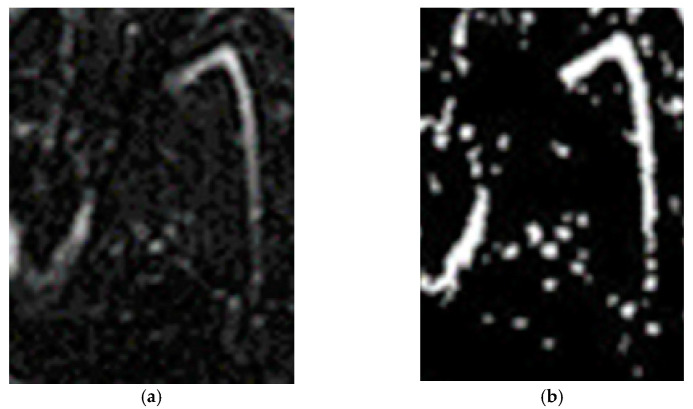

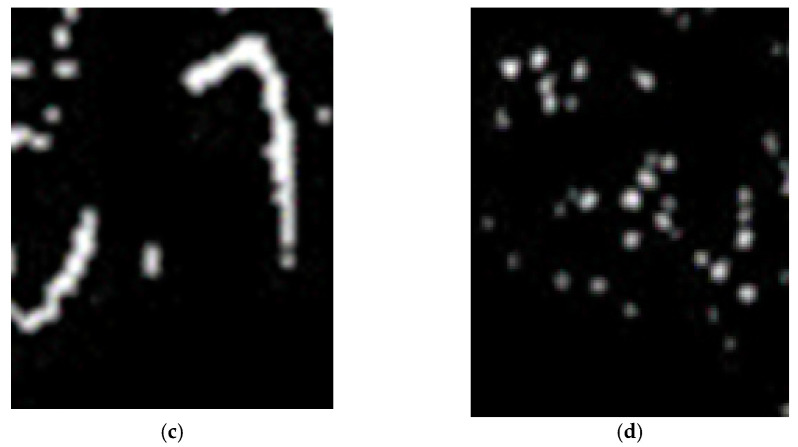
(**a**) Shows the 3D slice of the mouse calf region; (**b**) shows the binary version of (**a**); and (**c**) shows the results of morphological operation on (**b**); and (**d**) shows the final results of extracted IO-NPs.

Figure 5a shows a slice of the 3D image of the mouse calf; Figure 5b shows the binary version of Figure 5a by using Otsu’s binary algorithm. In this binary image, calf structure and IO-NPs are present. Figure 5c shows the results of morphological operations on Figure 5b where there are no IO-NPs, only the calf structure is present. In order to extract the IO-NPs from Figure 5b, a subtraction between Figure 5b,c was performed; the results are shown in Figure 5d. The cloud of white pixels inside Figure 5d show the concentration of IO-NPs attached to the drug to be delivered to the mouse calf.

**Figure 6:**
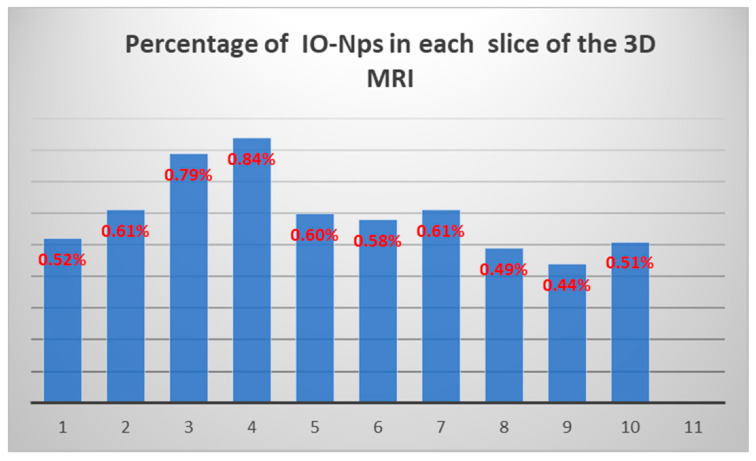
Percentage of IO-NPs of 10 slices from 3D MRI image.

The same procedure in Figure 5 is repeated on several slices of the 3D model of the mouse calf and the extracted IO-NPs represented by white pixels are counted in each slice. The results shown in Table 1 are the sum of white pixels in each slice representing the IO-NPs clusters; for the first image, the total number of white pixels is 476 (considered IO-NPs). A small variation in percentage among the stack of images is noticed in Figure 6. The exact quantity of IO-NPs could not be accurately estimated due to the relatively low resolution of MRI images (50 micrometer per pixels) compared to the size of the IO-NPs (~11 nanometers in diameter). A follow-up analysis using electron microscopy could improve the precision of the drug delivery monitoring process.

## 4. Discussions

The resolution of the MRI images is about 50 × 50 × 50 μm^3^ per pixel. The average diameter of IO-NPs used in this experiment is about 11 nm. Each pixel may contain hundreds of IO-NPs. Counting the blue pixels in the ROI in each slice provides an estimation of the surface covered by NPs. Using transmission electron microscope (TEM) images with a resolution of about 10 angstroms per pixel (1 nm), an estimation of the average number of IO-NPs [37] inside the macrophage was provided, by calculating the surface of one IO-NP (πd^2^/4) with the average diameter of 11 nm.

It is clear that, from HR-MRI images, we cannot estimate the number of IO-NPs inside the ROI, like from the TEM images. Nevertheless, the visualization of the 3D distribution of the clusters of IO-NPs will help locate macrophages inside the ROI and provide clear proof about their existence in agglomeration. In this study, the IO-NPs help in seeing the macrophage locations using MRI images. As we know, macrophages are used as a bus for drug delivery and IO-NPs can help detect the macrophage locations inside the target organ using MRI imaging. The visualization and quantification improve the accuracy of drug delivery by estimating the amount of drugs received by the ROI (inflammation or tumors). It can also help to control the amount of drugs in the therapy of nanomedicine procedures and provide an accurate diagnosis in the early stage of cancer with a relatively small lesion dimension.

The results obtained in this work are improved when compared to the results obtained in [20], where the k-means algorithm is used, demonstrating better detection of IO-NPs. This is due first to the fuzzy clustering technics, which consider elliptical shapes for clusters rather than circular. Second, the noise reduction is better here because we are working on slices from the 3D model of the mouse leg rather than a slice from the MRI system, which reduces the false detection of noise pixels. Finally, the extraction method is improved by using morphological operations instead of using connected components as in [20]. Seeing the IO-NPs inside ROI in 3D MR imaging and approximating their concentration will certainly help in reducing the undesirable consequence of drugs on healthy tissue, in addition to providing a visual tool to control the drug delivery processes and open a promising novel research axis for nanomedicine applications. 

## 5. Conclusions

This paper describes a method for extracting clusters of IO-NPs pixels from grayscale HR-MRI images for drug delivery monitoring in lesions and tumors. The clusters of IO-NPs were extracted from the HR-MRI images of the mouse calf across many stages. After applying a median filter to the entire stack of 50 images for background correction and noise filtering, a FCM algorithm was used to segment the images into seven classes. The next step was to use Otsu’s binarization method to isolate the IO-NPs from the calf tissue and other structures. Finally, the slices were used to recreate a 3D volume that showed the distribution of IO-NPs across the entire ROI.

We can see the spread of clusters of IO-NPs within the ROI and measure the amount of macrophages banded to IO-NPs. 

The proposed method isolates clusters of IO-NPs, labeled macrophages, from mouse calf tissues, and displays them in a 3D space. The ability to see the 3D distribution of clusters of IO-NPs within the ROI assists in the diagnosis and treatment in some nanomedicine applications. This method of processing will undoubtedly aid researchers in the field of nanomedicine, especially in cancer, in optimizing diagnosis and treatment strategies, by ensuring drug delivery to the tumor using an MRI imaging device.

## Figures and Tables

**Table 1:** Displays numbers and percentage of white pixels, considered IO-NPs, in 10 slices from the 3D of MRI images.

Image Number	Number of White Pixels	Percentage of NPs %
1	476	0.52%
2	553	0.61%
3	711	0.79%
4	754	0.84%
5	537	0.6%
6	523	0.58%
7	542	0.61%
8	438	0.49%
9	397	0.441%
10	432	0.51%

## Data Availability

Not applicable.
